# Targeting Inflammatory Pathways in Cardiovascular Disease: The Inflammasome, Interleukin-1, Interleukin-6 and Beyond

**DOI:** 10.3390/cells10040951

**Published:** 2021-04-20

**Authors:** Peter Libby

**Affiliations:** Division of Cardiovascular Medicine, Department of Medicine, Brigham and Women’s Hospital, Harvard Medical School, Boston, MA 02115, USA; plibby@bwh.harvard.edu

**Keywords:** cytokines, thrombosis, myocardial infarction, ischemic heart disease, vascular biology, acute coronary syndromes, innate immunity

## Abstract

Recent clinical trials have now firmly established that inflammation participates causally in human atherosclerosis. These observations point the way toward novel treatments that add to established therapies to help stem the growing global epidemic of cardiovascular disease. Fortunately, we now have a number of actionable targets whose clinical exploration will help achieve the goal of optimizing beneficial effects while avoiding undue interference with host defenses or other unwanted actions. This review aims to furnish the foundation for this quest by critical evaluation of the current state of anti-inflammatory interventions within close reach of clinical application, with a primary focus on innate immunity. In particular, this paper highlights the pathway from the inflammasome, through interleukin (IL)-1 to IL-6 supported by a promising body of pre-clinical, clinical, and human genetic data. This paper also considers the use of biomarkers to guide allocation of anti-inflammatory therapies as a step toward realizing the promise of precision medicine. The validation of decades of experimental work and association studies in humans by recent clinical investigations provides a strong impetus for further efforts to target inflammation in atherosclerosis to address the considerable risk that remains despite current therapies.

## 1. Introduction

The literature now abounds with publications on inflammation in atherosclerosis. Yet, only a few years ago, this concept met with considerable skepticism in many quarters. Ultimately, the results of recent clinical trials have firmly established that inflammation participates in generating atherosclerotic events in humans. Understanding the roots of this success, and honing our interventions ever more selectively, requires knowledge of the biological bases of anti-inflammatory therapies. This review aims to furnish that foundation and evaluate critically the current state of anti-inflammatory interventions.

## 2. Cytokines

Much of the ferment regarding inflammation in atherosclerosis has emerged from the characterization and understanding the roles of cytokines in vascular biology. Cytokines, protein mediators of inflammation and immunity, participate in both innate and adaptive immunity. A prototypical example, interleukin (IL)-1 rests atop a hierarchy of innate immune signaling. Tumor necrosis factor (TNF), also known initially as cachexin, shares many actions with IL-1. Like IL-1, TNF merits a place at the apex of a cytokine signaling pathway. This review will focus heavily on IL-6, a cytokine with a complex signaling pattern, because it is becoming clinically actionable as a target in atherosclerosis therapy. On the adaptive axis, interferon-gamma (IFN-γ) contributes essentially to crosstalk between the adaptive and innate arms of immunity. Produced by activated T lymphocytes and other adaptive immune cells, IFN-γ signals to many cell types including mononuclear phagocytes, the foot soldiers of the innate immune response. Like IL-1 and TNF, IFN-γ also affects important functions of vascular endothelial and smooth muscle cells along with many other cell types of multiple organs. Each member of this cytokine trio can induce the expression of chemokines, protein mediators of directed migration of various classes of leukocytes [1]. The interplay of cytokine and chemokines and their receptors regulate major aspects of many acute and chronic inflammatory and host defense mechanisms.

The complex networks of cytokine signaling provide a rich tapestry of targets for intervention in disease. Yet, given their often essential roles in host defenses, cytokine inhibition can prove a double-edged sword. Effective blockade of major cytokines, particularly those situated proximally in inflammatory pathways, comes with the potential limitation of impairment of host defenses. Examples include the important role of IFN-γ in defending against intracellular infectious agents such as tubercule bacillus. Inhibition of TNF can impair tumor surveillance and predispose to the development of lymphomas. Blockade of IL-1β can increase viral and bacterial infections, indicating an important function of this central cytokine in combatting common infectious pathogens.

## 3. IL-1 Production and Responsiveness, and the Discovery of Arterial Autocrine/Paracrine Loops

**Figure 1:**
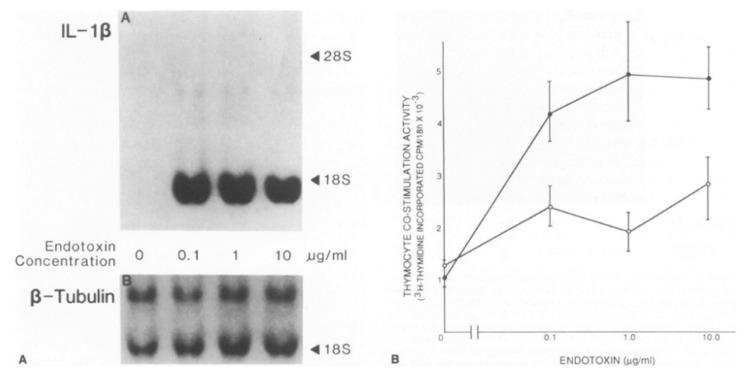
Vascular cells can produce IL-1. (**A**). This Northern blot shows induction of the messenger RNA encoding interleukin-1β by Gram-negative bacterial endotoxin. The top panel shows IL-1β transcript. The bottom panel shows β-Tubulin expression in a re-hybridization of the same blot. The left-hand panel (**B**) shows interleukin-1 activity as measured by thymocyte co-stimulation. The supernatants of the cultures of the cells probed for messenger RNA in the left-hand panel were analyzed for biological activity showing the induction of release of activity with incubation with endotoxin. Addition of polymyxin B, an LPS inhibitor, to the medium during exposure to LPS blunted the rise in thymocyte co-stimulatory activity. (open circles). *From Am J Path. Libby* et al. *1986, 124:179-185*.

**Figure 2:**
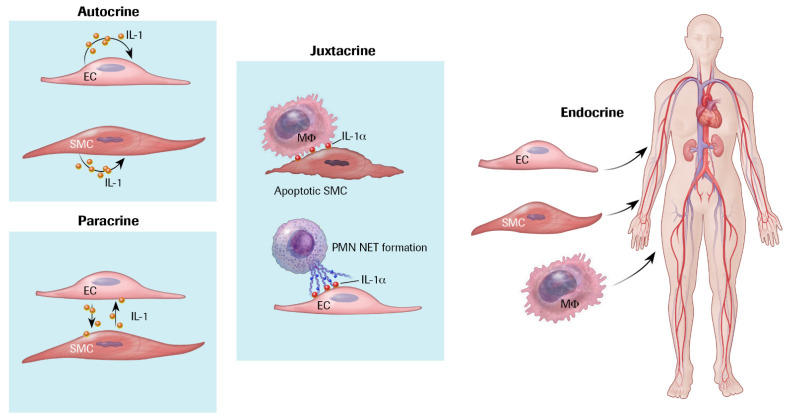
Cytokines can act through autocrine, paracrine, juxtacrine, or endocrine pathways. Cytokine signaling can operate in different ways. Autocrine signaling, shown on the upper left, can involve IL-1-induced IL-1 expression. Paracrine signaling denotes exchange of cytokines such as IL-1 between neighboring cells as exemplified here by endothelial cell and smooth muscle cell crosstalk. Juxtacrine signaling involves cell contact. Two examples pertinent to cytokine signaling in vascular pathophysiology include IL-1α expressed on the surface of smooth muscle cells that are activated or are undergoing death. IL-1α can signal to macrophages by contact. IL-1α also associates with neutrophil extracellular traps (NETs) and can activate endothelial cell pro-inflammatory functions such as adhesion molecule expression and tissue factor generation in a manner that requires contact. Finally, the circulation can carry secreted cytokines to distant organs, denoted endocrine signaling. For example, IL-6 secreted by vascular cells as well as leukocytes can circulate to encounter hepatocytes and trigger the acute phase response as shown in Figure 3.

Initially, investigators viewed IL-1 as mediating signaling between leukocytes, hence its designation “inter-leukin.” My laboratory made the initially controversial discovery that endogenous vascular cells, the endothelium and smooth muscle, could produce IL-1 (Figure 1) [2,3]. This discovery laid the foundation for the possibility of autocrine and paracrine signaling in blood vessels (Figure 2). Early on, we defined the inducible expression of the genes that encode both isoforms of IL-1, α and β. Our initial experiments used Gram-negative bacterial endotoxin (the lipopolysaccharide endotoxic principle, LPS) as a stimulus. At the time, we regarded LPS as laboratory artifice in regard to vascular cell activation. Yet, the burgeoning interest in the intestinal microbiome, and impaired epithelial barrier function in certain diseases such as HIV/AIDS, renders our early experiments much more relevant to pathophysiology than we conceived at the time.

Cytokines can signal locally or at a distance (Figure 2). As the activated vascular cells release interleukin-1β via what we now recognize as the gasdermin D export pathway, this IL-1 isoform can act extracellularly in an autocrine or paracrine manner. Interleukin-1α generally associates with the cell membrane positioning it for juxtracrine signaling by direct contact with responding cells. Dead or dying cells can also release interleukin-1α, a cytokine implicated in the senescence-associated secretory response. Since our initial description of inducible cytokine expression by vascular wall cells, hundreds if not thousands of subsequent publications have amplified our initial discoveries. Work from our own and many other laboratories has localized entire families of these mediators in human atherosclerotic plaques as well as their companion pro-inflammatory cytokines [4,5,6].

## 4. Autoinduction of IL-1: A Positive Feedback Loop

**Figure 3:**
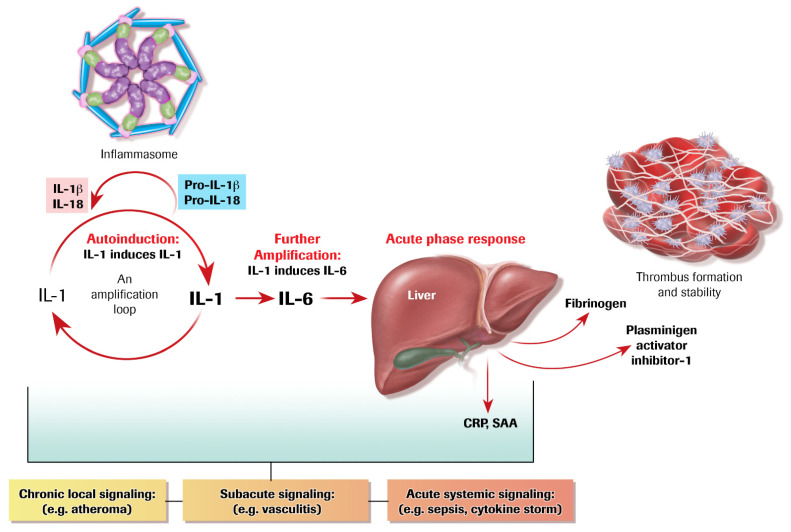
Amplification loops in cytokine signaling. IL-1 induces its own gene expression, auto-induction as shown on the left-hand of this figure. IL-1 can in turn induce IL-6 from many cell types, an amplification loop. IL-6 triggers the acute phase response in hepatocytes which augments fibrinogen, the precursor of clots, and plasminogen activator inhibitor-1 (PAI-1), proteins that favor clot formation and resist fibrinolysis. The acute phase reactants C-reactive protein (CRP) and serum amyloid A (SAA) can serve as biomarkers of the inflammatory response. The inflammasome can process the inactive forms of IL-1β and IL-18 to their biologically mature forms instigating this inflammatory cytokine cascade. This series of amplification steps can occur locally in a chronic disease such as atherosclerosis, more acutely locally in many diseases including the vasculitides. The positive feedback loop can lead to cytokine storm associated with sepsis or acute viral infections such as SARS-CoV-2.

**Figure 4:**
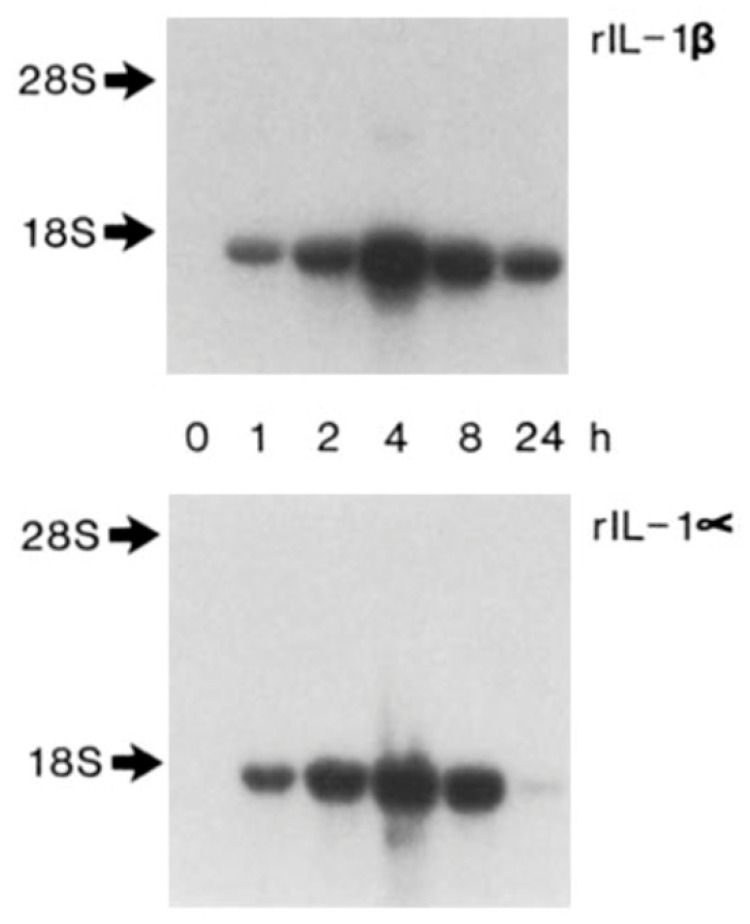
Autoinduction of IL-1 amplifies inflammatory signaling. IL-1 induces IL-1 gene expression. Either recombinant IL-1β (top) or recombinant IL-1α (bottom) induced the messenger RNA encoding IL-1β. Messenger RNA as shown by Northern blotting. Either isoform of IL-1 induced the biological activity as well (not shown.) The concentration of IL-1β was 100 ng/mL and IL-1α was 10 ng/mL. *From J Exp Med 165:1316-1331; 1987.*

Soon after defining the ability of cells other than leukocytes to produce IL-1, my group discovered that IL-1 can induce its own gene expression in vascular endothelial and smooth muscle cells (Figure 3 and Figure 4) [7,8]. Joining forces with Charles Dinarello, who led the heroic purification and cloning of the cDNA that encodes interleukin-1β [9], we described the ability of IL-1 to induce its own gene expression in human mononuclear phagocytes and in rabbits in vivo [10]. These observations established the operation of positive feedback amplification loops that could operate in the wall of blood vessels as well as during many innate immune responses in the arterial wall and other organs (Figure 3). This phlogistic potential could lead to untrammeled overexpression not only of IL-1, but also of other downstream mediators that it triggers, both protein and lipid.

These mediators induced by IL-1 include not only many other cytokines, but also lipid-signaling molecules including prostanoids such as prostaglandin E_2_, the major mediator of fever [11]. Such phenomena operate not only locally within inflammatory lesions, including the atherosclerotic plaque, but also systemically (Figure 2). Indeed, in sepsis, either bacterial or viral, IL-1-induced IL-1 may amplify the systemic responses associated with multi-organ system dysfunction. Overwhelming viral or bacterial infections can lead to a cytokine storm in which IL-1 autoinduction may play a proximal role. This feed-forward loop doubtless contributes to the end stage of COVID-19 during infection with SARS-CoV-2, which often involves adult respiratory distress syndrome and microvascular injury and failure of multiple other organs including the kidneys, the liver, and the myocardium [12]. Advanced COVID-19 provides a particularly poignant example of cytokine storm, which may result from IL-1-induced IL-1 [12,13,14].

In biological signaling, counter regulatory mechanisms often operate to antagonize such positive feedback loops. In the case of IL-1, its endogenous receptor antagonist, IL-1ra, furnishes an example of another member of the IL-1 family [15,16]. Many of the same stimuli that induce the agonist isoforms of this cytokine induce the antagonist, IL-1ra. This negative feedback loop can moderate the consequences of IL-1-induced IL-1 production. Indeed, the IL-1 receptor antagonist has become a drug which has proven useful in certain auto-inflammatory diseases and acute and chronic cardiovascular conditions [17,18].

## 5. IL-1 Induces IL-6: Even More Amplification

**Figure 5:**
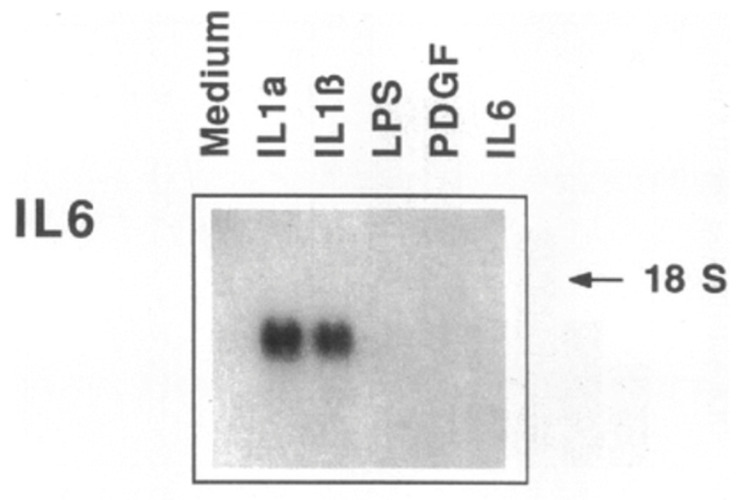
IL-1 induces IL-6, a further amplification of cytokine signaling. This Northern blot shows the expression of the messenger RNA encoding interleukin-6 in human smooth muscle cells incubated with interleukin-1 isoforms, bacterial lipopolysaccharide (LPS), platelet-derived growth factor (PDGF) or IL-1 itself. IL-1 strongly induces IL-6 messenger RNA and in other experiments not shown induced IL-6 protein synthesis as shown by metabolic labeling and biological activity as determined by thymidine incorporation by B9 cells. *From J Clin Invest 85:731-738, 1990.*

In the late 1980s, our laboratory described IL-1-induced IL-6 expression (Figure 3 and Figure 5). We found that in human vascular endothelial and smooth muscle cells, stimulation with either IL-1α or β could elicit the production and release of copious amounts of IL-6. This second level of amplification began to define a pathway. IL-6 is a soluble cytokine that can act at a distance. Thus, in addition to the autocrine, paracrine, and juxtracrine signaling described above, IL-6 may participate in “endocrine” signaling. Indeed, we dubbed IL-6 a “messenger” cytokine on the basis of its ability to reach the hepatocyte and trigger the acute phase response (Figure 3). We initially focused on IL-1 regulation of IL-6 because of IL-6’s known property of activating the acute phase response (Figure 3).

## 6. The Acute Phase Response Lies Downstream of IL-1 and IL-6

During systemic inflammatory responses, IL-6 triggers an alteration in the program of protein synthesis in liver cells. The acute phase response diverts some of the protein production by hepatocytes from “housekeeping” proteins such as albumin to a series of proteins implicated in host defenses. Among the acute phase reactants, fibrinogen, the precursor of clots, and plasminogen activator inhibitor-1 (PAI-1) figure prominently (Figure 3). Boosted production of these proteins can help to staunch bleeding following a wound by augmenting clot formation and impeding endogenous fibrinolytic pathways. While important in host defenses, these alterations in hemostasis could clearly contribute to the thrombotic complications of atherosclerosis.

Other proteins produced as part of the acute phase response included C-reactive protein (CRP) and serum amyloid A (SAA) (Figure 3). CRP derived its name from the ability to interact with capsular polysaccharides of the Pneumococcus. CRP can thus participate in host defenses against such bacterial pathogens. SAA, a principal pentraxin product of the acute phase response in mice as well as humans, may promote the remodeling of high-density lipoprotein associated with impaired ability to engage in reverse cholesterol transport [19]. HDL contains many proteins including an LPS binding protein. Thus, HDL can serve as a sink for LPS, potentially playing a counter-regulatory role during endotoxemia and the attendant consequences of bacterial sepsis. CRP, measured with a highly sensitive assay (hsCRP) can predict first ever and recurrent cardiovascular events, rendering it a useful biomarker for cardiovascular risk assessment [20]. Therapy guided by CRP concentration provides an example of precision allocation of an anti-inflammatory intervention as well [21].

## 7. The Inflammasome Family: Proximal Activators of Pro-Inflammatory Cytokines

**Figure 6:**
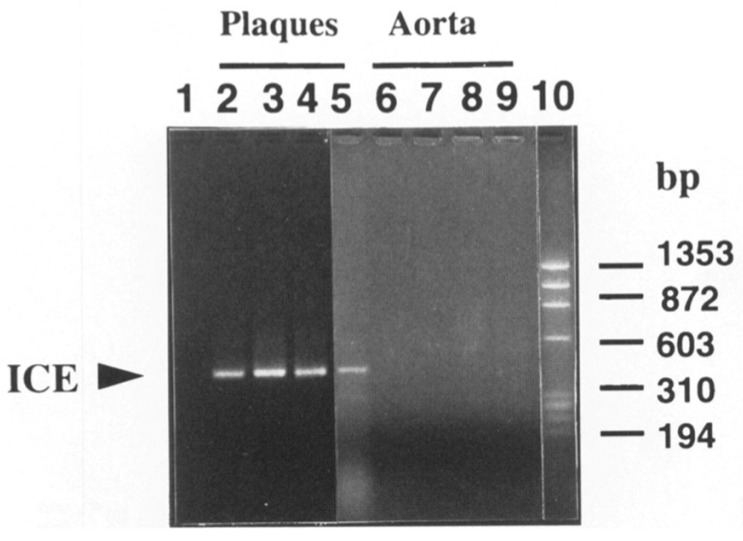
Inflammasomes operate in human atherosclerosis. Human atherosclerotic plaques express the messenger RNA encoding caspase-1 or interleukin-1β converting enzyme (ICE). Lane 1 shows an RT-PCR reaction without template. Lanes 2–5 represent RNA from extracts of human atherosclerotic plaques. Lanes 6–9 depict analysis of extracts of a normal aorta as control. Lane 10 shows size markers. The protein product was also demonstrated in the paper cited [22]. *From Geng and Libby. Am J Path 147:251-266; 1995.*

Like many proteins pivotal in biological control, the initial translational products of IL-1β and its cousin IL-18 require proteolytic processing to attain biological function. IL-1α depends less on limited proteolytic activation to exert its biological actions (Figure 3, upper left). The major protease implicated in processing the precursors of IL-1β and IL-18, caspase-1, founded the caspase family of aspartyl proteinases. We localized caspase-1 mRNA and protein in the human atherosclerotic plaque in 1995 (Figure 6) [22]. We demonstrated the ability of a pro-inflammatory cytokine that we had implicated in inflammatory signaling within atheromata, CD40 ligand (CD154), as an activator of the processing of pro-IL-1β to the active cytokine in human vascular cells [23]. Simultaneous with this work in our own laboratory, the late Jürg Tschopp discovered that caspase-1 is the machinery protease in the inflammasome, a family of macromolecular multi-component protein structures that respond to danger signals including pathogen-associated molecular patterns (PAMPs) and damage associated molecular patterns (DAMPs) [24].

Many stimuli relevant to atherosclerosis can activate inflammasomes and promote the processing of pro-IL-1β to its active form. Such stimuli include cholesterol crystals, hypoxia, and disturbed flow [25,26,27,28]. Beyond cholesterol, apolipoprotein CIII, a modulator of triglyceride metabolism, and implicated by strong clinical and human genetic evidence in inciting atherosclerotic risk, can also stimulate inflammasome activation [29]. Inflammasomes generally require multiple signals for activation. In this manner, the inflammasome family proximally activates the key pro-inflammatory cytokine IL-1β unleashing all of its downstream actions profiled above. The inflammasome family includes a number of members including two that participate particularly in atherogenesis as delineated in studies in mice. The NLRP3 inflammasome, co-activated by cholesterol crystals, has received much attention in atherosclerosis research [18,30,31,32]. Rendering mouse experiments relevant to clinical atherosclerosis, human coronary artery atheroma that contain cholesterol crystal detected in vivo by intravascular imaging also exhibit imaging evidence for harboring morphologic characteristics associated with propensity to provoke acute coronary events [33]. Moreover, a common genetic variant that elevates NLRP3 expression in human monocytes, associates with higher hsCRP concentrations and coronary artery disease prevalence [34]. Recent evidence also implicated the AIM2 inflammasome in experimental atherosclerosis accelerated by a mutation in Janus Kinase 2 (*JAK*2^V617F^) that causes clonal hemopoiesis of indeterminate potential, a condition that drives atherosclerotic events independent of traditional risk factors [35].

## 8. Targeting Inflammasomes: Colchicine and Small Molecules

Given the proximal role of the NLRP3 inflammasome in auto-inflammatory diseases and in the response to pathogens and tissue damage, it is not surprising that there is considerable interest in small molecules that inhibit this macromolecular assembly. A number of programs in the biotechnology and pharmaceutical sector have produced inhibitors of the NLRP3 inflammasome [31,36]. Their availability has heightened interest in the clinical evaluation of inflammasome inhibitors. One might harbor concern about inhibition of such a proximal step in host defense pathways. Yet, the very existence of various family members indicates that the redundancy of inflammasomes could provide compensation for blockade of one family member or the other. Thus, conceptually targeting selectively various members of the inflammasome family could mitigate the downstream consequences of inflammasome activation without necessarily impeding the family’s role in host defenses.

A natural product small molecule, colchicine, derived from the autumn crocus plant, has seen use for centuries as an anti-inflammatory. In particular, colchicine has a major role in treating acute gouty arthritis attacks (podagra). Monosodium urate crystals clearly provoke gout attacks, and the inflammasome mediates this disease. Colchicine has become a primary therapeutic for chronic auto-inflammatory conditions including familial Mediterranean fever, as pioneered by a team that included Sheldon M. Wolff, Charles A. Dinarello, and Anthony Fauci [37]. Colchicine has entered the mainstream of cardiovascular practice as a treatment for pericarditis [38]. Its widespread use for this indication has lessened our dependence on glucocorticosteroids, a therapy that has proven very difficult to wean in the management of recurrent pericarditis.

Colchicine acts by interfering with the assembly of microtubules. Colchicine can thus interfere with the assembly of the multiple components that comprise inflammasomes. One of colchicine’s putative mechanisms of action, in addition to impairing leukocyte locomotion, involves inflammasome inhibition [39]. Recent studies have demonstrated the efficacy of colchicine in reducing recurrent cardiovascular events in patients with atherosclerosis. The initial small-scale LoDoCo study instigated interest in this regard [40]. The large-scale COLCOT trial in patients enrolled soon after an acute coronary syndrome showed a reduction in recurrent major adverse cardiovascular events including revascularization [41]. The LoDoCo2 study in patients in the chronic phase of coronary artery disease showed similar efficacy [42]. These studies together not only provide strong support for the inflammatory basis of atherosclerosis, but also point to targeting the inflammasome as a promising therapeutic strategy in this disease. Initial proteomic analysis of LoDoCo2, however, did not show a pattern of altered protein expression compatible with inflammasome inhibition providing a major contribution to the clinical efficacy [43]. The question of the mechanism of colchicine’s action including and beyond the inflammasome merits much closer study. Moreover, the safety of colchicine in individuals with impaired renal function, infection risk, and the gastrointestinal tolerability in broader populations will require ongoing assessment.

## 9. Targeting IL-1β

Antagonizing the action of IL-1β has generated considerable interest in cardiovascular disease. Approaches to inhibition of IL-1β action include administration of the endogenous receptor antagonist IL-1ra, a decoy (rilonacept), other traps, and a monoclonal antibody strategy. Small clinical trials have indicated benefit of treatment with IL-1ra in reducing the inflammatory response after acute coronary syndromes in the MRC IL-1 study [44] and a series of studies carried out by the group of Antonio Abbate targeting myocardial infarction and heart failure [18]. The recent RHAPSODY study established the efficacy of rilonacept in the management of recurrent pericarditis [45].

A specific anti-IL-1β antibody, canakinumab, has received approval as an orphan drug for treating rare auto-inflammatory conditions caused by mutations that yield a gain of function of the NRLP3 inflammasome. These diseases include Muckel–Wells syndrome and cryopyrin-associated periodic syndrome. The Canakinumab Anti-inflammatory Thrombosis Outcomes Study (CANTOS) enrolled over ten thousand individuals who had sustained an acute myocardial infarction at least thirty days before enrollment and had evidence of persistent inflammation despite treatment with a full panel of guideline-directed medical therapy including high-intensity statin treatment [46]. Selection of individuals with a hsCRP greater than median for the population (>2.0 mg/L) identified patients with persistent inflammation despite standard of care treatments. Canakinumab, administered four times yearly, reduced recurrent major adverse cardiovascular events defined as myocardial infarction, stroke, or cardiovascular death [47]. This large-scale clinical trial was the first to demonstrate efficacy of an anti-inflammatory intervention in patients at risk for atherosclerotic events. The magnitude of reduction in events in these well-treated patients resembled numerically the extent of benefit produced by the biological therapies that lower low-density lipoprotein by inhibition of proprotein convertase subtilisin/kexin type 9 (PCSK9) [48,49].

The individuals treated with canakinumab had a slight but statistically significant increase in infections including those that yielded fatal outcomes. The study showed overall neutrality for mortality because of a striking reduction in incident cancer and death due to cancer, primarily driven by lung cancer [50]. In the individuals who responded well to canakinumab by reducing hsCRP by greater than 50%, a pre-specified on-treatment analysis showed that the intervention reduced cardiovascular and all-cause mortality greater than 30% [51]. A variety of sensitivity analyses affirmed these conclusions of the on-treatment analysis, countering possible confounding. Thus, three different interventions that inhibit IL-1β have shown efficacy in clinical trials investigating cardiovascular endpoints. Of note, the IL-1 receptor antagonist and rilonacept block both the IL-1β and IL-1α isoforms of this pro-inflammatory cytokine.

## 10. Targeting IL-1α

**Figure 7:**
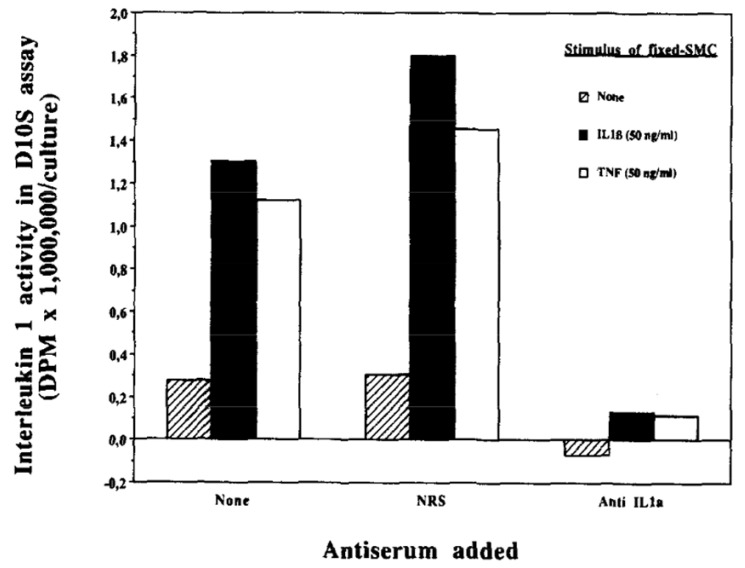
IL-1α activates smooth muscle cells via juxtacrine signaling. Smooth muscle cell monolayers were lightly fixed with paraformaldehyde after stimulation with IL-1β or tumor necrosis factor. A responder layer of smooth muscle cells was seeded above the fixed smooth muscle cells. Thymidine incorporation into D10S cells assessed IL-1 activity. The responder cells also elaborated IL-6 in other experiments not shown. *From Exp Cell Res 198:283-290;1992.*

Despite its biochemical similarity, IL-1α and IL-1β differ substantially in their cell biology and likely participation in pathophysiologic responses [18]. As noted above, IL-1α depends less on proteolytic cleavage to exert its pro-inflammatory actions. Thus, the alpha isoform of IL-1 does not depend substantially on caspase-1 processing and the inflammasome family. IL-1α also probably participates primarily in paracrine or juxtracrine signaling as it associates with cell surface membranes and release from dead or dying cells (Figure 2). Indeed, we showed that juxtacrine signaling due to IL-1α can activate human smooth muscle cells, illustrating the operation of mechanisms for a local juxtacrine positive feedback loop in vascular disease (Figure 7) [52].

In the context of cardiovascular disease, and atherothrombosis in particular, IL-1α and IL-1β may subserve distinct functions [53,54,55]. For example, the ability of neutrophil extracellular traps (NETs) to elicit inflammatory responses from human endothelial cells depends on the alpha isoform rather than IL-1β (Figure 2) [56]. The granulocyte serine proteinase cathepsin G can process IL-1α, augmenting its pro-inflammatory action. NET formation participates in many forms of vascular thrombosis ranging from deep venous thrombosis to acute coronary syndromes, and likely some of the thrombotic complications of SARS-CoV-2 infection, including those that affect the cardiovascular system.^12^ A monoclonal antibody that neutralizes IL-1α can mitigate early atherosclerosis in hypercholesterolemic mice and can reduce experimental stroke [57,58]. Clinical trials have not yet established a role for IL-1α inhibition in cardiovascular events. Yet, a substantial body of evidence indicates the potential of IL-1α inhibition in reducing thrombotic complications of atherosclerosis and in cardiovascular conditions associated with cell death such as myocardial ischemic injury.

## 11. Targeting Interleukin-6

**Figure 8:**
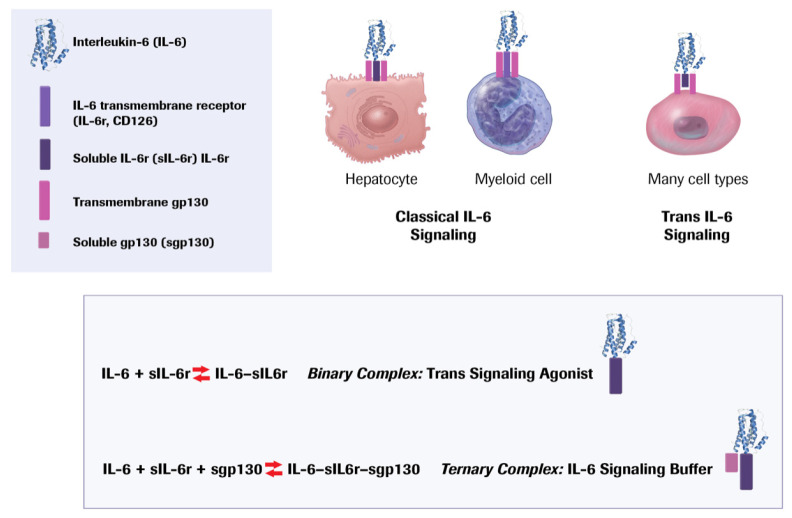
IL-6 signaling is complex and subject to multiple levels of regulation. As described in the text, classical IL-6 signaling involves binding of the ligand to the membrane bound IL-6 receptor (CD 126.) Hepatocytes and leukocytes express this receptor, which associates with gp130 to initiate transmembrane signaling. However, the surface IL-6 receptor can also undergo limited proteolytic cleavage by ADAM 17, releasing a soluble form of this receptor. This soluble IL-6 receptor can bind to the IL-6 and form a binary complex. This binary complex can bind to gp130, which is expressed ubiquitously, effecting trans IL 6 signaling to multiple cell types. A soluble form of gp130 can combine with the binary complex, forming a ternary complex that sequesters IL-6 bound to its receptor, providing a buffer for trans IL-6 signaling. A common variant in the IL-6 receptor can promote its shedding, depleting the hepatocyte or leukocyte surface of this receptor (CD126), limiting classical signaling, but increasing formation of the binary complex by mass action, and thus favoring trans IL-6 signaling.

IL-6 signaling can occur through its cell surface receptor IL-6Rα (CD126) expressed on hepatocytes and leukocyte subclasses, particularly myeloid cells and αβ T cells, as well as B1α lymphocytes [59]. This pathway, denoted “classical,” may exert anti-inflammatory actions (Figure 8) [60]. Yet, in view of IL-6 stimulation of the hepatocyte acute phase response, this classical pathway might well augment the formation and persistence of clots due to the induction of fibrinogen and PAI-1 [61].

The IL-6 receptor can undergo proteolytic cleavage, releasing a soluble form that can combine with the co-receptor for IL-6, glycoprotein 130 (gp130). Many cell types express gp130. Thus, the complex of the soluble receptor and the IL-6 ligand can activate a variety of cell types, a pathway known as “trans” signaling (Figure 8). Many ascribe a pro-inflammatory role to trans IL-6 signaling. The binary complex of gp130 and the soluble receptor may limit trans signaling by competing for cell surface bound gp130.

Several strategies exist that target IL-6 signaling. Ziltivekimab neutralizes IL-6 ligand. Tocilizumab and sarilumab block the IL-6 receptor. These interventions should limit both classical and trans-signaling by IL-6. A soluble gp130 protein fused with the Fc portion of IgG (olamkicept) can inhibit trans-signaling by IL-6 [62].

Administration of IL-6 to ApoE-deficient mice can augment plaque burden without alteration in cholesterolemia [63]. Some studies show that IL-6 deficiency in atherosclerosis-prone apolipoprotein E-deficient mice can actually display increased atherosclerosis. *ApoE^−/−^ Il6^−/−^* mice in one study consumed a chow diet (i.e., not supplemented with fat or cholesterol), and had a substantial increase in plasma cholesterol concentration compared to mice wildtype for *Il6* [64]. Yet, inflammatory cell content of the atherosclerotic plaques decreased in the absence of IL-6. This apparent contradiction requires further exploration. Another study that compared *Il6^+/−^* mice to *Il6^−/−^ Apoe^−/−^* mice and showed increased atherosclerosis in the homozygous vs. heterozygous *Il6*-deficient mice, accompanied by an increased blood cholesterol level [65]. The selective inhibition of trans-IL-6 signaling by administering an excess of a soluble gp130 protein fused with the Fc portion of IgG can reduce experimental atherosclerosis in *Ldlr*-deficient mice [66]. Yet, hepatocyte selective deletion of gp130, which should mute putatively anti-inflammatory classical IL-6 signaling, reduces atherosclerosis [67]. The diverse experimental protocols used in such mouse experiments, and the confounding effects of alterations in lipid profiles effected by the manipulations of IL-6 action temper the conclusions one can draw from this contradictory and confusing literature [68,69,70].

Observations in humans help to place the disparate experimental results into perspective. About 40% of the population has a variant in the IL-6 receptor gene, IL6Rp.Asp358Ala. This variant favors the limited proteolytic cleavage of the IL-6 receptor releasing the soluble form while diminishing the availability of the receptor on the surface of hepatocytes and myeloid cells. Thus, individuals with this variant have reduced functioning of classical IL-6 signaling to leukocytes and liver cells. People with this variant additionally have reduced hsCRP, indicating a lower overall inflammatory burden and in a gene dosage-dependent manner also show reduced cardiovascular risk [71,72]. In humans, independent large-scale Mendelian randomization studies have yielded concordant results, indicating a net benefit for atherosclerotic outcomes of interference with IL-6 function. Clinical data affirm the relationship between IL-6 signaling and cardiovascular events [73]. Higher circulating levels of IL-6 associate with increased cardiovascular risk in large-scale clinical studies [74,75,76]. Post-acute coronary syndrome risk also relates to IL-6 concentrations in plasma [77,78,79,80]. Patients who have sustained a myocardial infarction have lower plasma concentrations of the endogenous antagonist of IL-6, soluble gp130, than matched individuals who have not suffered an acute coronary syndrome, but have higher concentrations of soluble IL-6 receptor that should favor trans-signaling [81]. Approaches to assessing IL-6 trans-signaling have used calculations of the “binary complex” of IL-6 and its soluble receptor (putatively pro inflammatory) and the “ternary complex” of IL-6, the soluble receptor, and the endogenous “antagonist,” soluble gp130. These computations require extrapolation of assumptions about binding constants assessed in vitro to the much more complex milieu of circulating blood. A greater abundance of the binary complex relative to the supposedly inactive ternary complex associates with greater risk of ischemic cardiovascular events [82].

Individuals subject to heightened cardiovascular risk due to clonal hematopoiesis arising from somatic mutations in *DNMT3a* or *TET2* with the genetically-determined decrement IL-6 signaling due to the IL6Rp.Asp358Ala variant have reduced risk of cardiovascular events [83]. These human genetic data provide very strong evidence supporting an overall pro-inflammatory role for IL-6 signaling. These findings also call in question the relevance to humans of the experimental observations that implicate IL-6 signaling as an anti-inflammatory pathway. Both human observations and mouse experiments implicate IL-6 in abdominal aortic aneurysmal disease, a condition commonly associated with atherosclerosis [84,85]. Clinical trials using tocilizumab, a monoclonal antibody that neutralizes IL-6, show lower inflammation in individuals with acute coronary syndromes, compatible with a beneficial effect of overall IL-6 inhibition in patients with coronary artery disease [86,87,88]. However, tocilizumabs increase atherogenic lipoproteins in humans [89,90]. As IL-6 lies distal to IL-1β (Figure 3), it may reduce cardiovascular events with less impairment of host defenses than IL-1β which inhibits inflammatory signaling and thus host defenses upstream of IL-6.

## 12. Targeting TNF

Tumor necrosis factor, like IL-1, exists in several isoforms. TNF, initially called TNF-α or cachexin, shares many properties with IL-1 and signals through its own set of surface membrane receptors [91,92]. TNF receptor 1 is involved in signaling of apoptosis and inflammation, whereas TNF receptor 2 primarily promotes inflammation [93,94]. Numerous biological pharmaceuticals can inhibit TNF signaling including neutralizing antibodies and a decoy soluble receptor including one that is a chimeric molecule linked to human IgG_1_ molecule. The use of such biologicals as a therapy for inflammatory diseases such as rheumatoid arthritis has proven very effective. Yet, in the cardiovascular arena, TNF antagonism has certain drawbacks [95]. Although TNF concentrations increase in patients with heart failure, and might contribute to cardiac cachexia, studies that have targeted TNF-α have not shown benefit in heart failure. Indeed, at a higher dose one such study showed a signal for hazard [96]. These findings have diminished enthusiasm for exploration of TNF inhibition in patients with atherosclerosis, a condition commonly associated with heart failure or risk of developing heart failure [90,97].

## 13. Targeting IL-18

IL-18 shares many properties with IL-1β. IL-18 is activated by the NLRP3 inflammasome. IL-18 produces many similar effects on vascular and other cells. Human atherosclerotic plaques contain the cytokine and its receptors. IL-18 elicits a number of functions associated with aggravation of atherosclerosis [98,99]. Strategies for inhibiting IL-18 have shown diminution in experimental atherosclerosis. We discovered an alternative receptor for IL-18, a sodium-chloride co-transporter [100]. This may be a first instance of an ion transporter as a receptor for a pro-inflammatory cytokine. We found in apolipoprotein E-deficient mice that lack of both the IL-18 receptor and the sodium-chloride co-transporter could limit atherosclerosis lesion development, but not single deficiency of either protein. Thus, IL-18 has interest as a target in inhibiting atherosclerosis. IL-18 is also involved in adipocyte signaling and the metabolic syndrome, indicating that its inhibition might have broader cardioprotective effects. In the IL-1β inhibition study CANTOS, canakinumab limited IL-6 concentrations in a dose-dependent manner. However, the blockade of IL-1β did not reduce IL-18 concentrations, indicating a selective effect of canakinumab of one but not the other major substrates for caspase-1 and activation by the NLRP3 inflammasome [75].

## 14. Targeting Nuclear Factor-Kappa B (NF-κB)

TNF, IL-1 isoforms, and a number of other pro-inflammatory cytokines ultimately regulate inflammatory gene expression by activation of nuclear factor-kappaB [101]. The signaling pathway for NF-κB activation has been elucidated in great detail, revealing a number of potential targets for intervening on this central hub of pro-inflammatory signaling [102]. These interventions include proteasome inhibitors that can block the degradation of the endogenous inhibitor IκBα [103]. High concentrations of salicylates, classical anti-inflammatory drugs, can inhibit NF-κB. There are a number of small molecule inhibitors that target the NF-κB signaling pathway. NF-κB can either protect against apoptosis or promote this process of programmed cell death depending on the experimental conditions. Given the essential role of NF-κB in host defenses and its role in regulating apoptosis, concerns regarding impairment of host defenses and potential emergence of malignancies have limited enthusiasm for targeting this key transcriptional mechanism in inflammation.

## 15. Targeting Adaptive Immune Responses

This review focuses on cytokines and innate immune response modification. Yet, in the context of atherosclerosis, a number of manipulations of adaptive immunity merit investigation [104,105]. Several strategies to vaccinate against a component of low-density lipoprotein to protect against atherosclerosis have undergone exploration [106,107,108]. While many pre-clinical studies have proven promising, vaccination strategies have not yet shown success in clinical trials. B cell subtypes, prominently B2 lymphocytes and mediators associated with B cell activation, might aggravate atherogenesis [109]. Yet, in mice neutralization of B cell-activating factor (BAFF) can augment experimental atherosclerosis despite reducing B2 cell numbers [104]. Rituximab, an anti-CD26 antibody, can deplete B cells and is widely used as an immunosuppressive agent in a number of clinical contexts. Study of the use of rituximab in atherosclerosis, and its complications, is underway [110]. Concerns regarding impairment of host defenses require careful scrutiny in this regard. Regulatory T cells that produce transforming growth factor-beta (TGF-β) can exert anti-inflammatory actions [111]. Low-dose interleukin-2 administration to humans can boost regulatory T cells [112]. Preliminary clinical studies examining this strategy in patients with acute coronary syndromes are underway. Other strategies involve manipulating heat shock protein 27 (hsp27) which can serve as a biomarker of cardiovascular events but may also limit atherosclerosis. Vaccination of ApoE-deficient mice with the murine ortholog of hsp27, hsp25, can reduce experimental atherosclerosis [113].

A number of other cytokines have received consideration as targets in atherosclerosis, notably IL-23 [90,97]. Yet, studies on blockade of IL-23 and of the IL-17 axis have shown signals for increased cardiovascular risk, limiting enthusiasm for their further exploration in atherosclerosis therapy [90]. Interleukin-17, the signature cytokine of Th17 cells, can exert many pro-inflammatory functions. The experimental literature, however, does not show consistent benefit of loss of IL-17 function in experimental atherosclerosis [90,97].

A number of other anti-inflammatory strategies have undergone clinical investigation. Various phospholipase inhibitors have failed to reduce cardiovascular events despite promising pre-clinical data. The SOLID and STABILITY studies did not show benefit of lipoprotein-associated phospholipase A_2_ in patients with acute or chronic atherosclerotic disease [114,115]. Inhibition of p38 map kinase (p38MAPK), although having a strong fundamental rationale as a target in atherosclerosis, failed to reduce events in LATITUDE [116].

## 16. Precision Medicine—A Path Forward?

We have become victims of our own success in cardiovascular medicine. Currently effective therapies for atherosclerosis and heart failure have raised the bar for clinical trialists to demonstrate the efficacy of additional therapeutic strategies. Currently, studies designed to test the ability of anti-inflammatory agents to improve cardiovascular outcomes require background treatment with highly effective therapies, reducing the event rates, increasing the necessary sample size and/or the duration of studies. These considerations present considerable obstacles to the development of novel cardiovascular therapies. Fortunately, anti-inflammatory interventions act orthogonally to the conventional lipid-lowering and anti-thrombotic therapies for atherosclerosis and neurohumoral blockade in heart failure. Yet, the need to demonstrate incremental benefit over established therapies remains an obstacle to the clinical development of anti-inflammatory interventions for atherosclerosis.

One approach to addressing this conundrum involves using biomarkers to select individuals at particularly high risk for cardiovascular complications driven by inflammation. The use of C-reactive protein as an entry criterion in the Justification for the Use of statins in Prevention: An Intervention Trial Evaluating Rosuvastatin (JUPITER) in primary prevention and in CANTOS in secondary prevention no doubt enhanced the favorable outcomes of these studies [21,47].

Beyond CRP, what criteria could identify individuals at particular risk due to specific inflammatory pathways? Imaging biomarkers have considerable limitations as enrollment criteria for clinical trials. For example, coronary artery calcium scores, while they indubitably predict cardiovascular risk, do not appear modifiable by interventions thus far tested. Moreover, coronary artery calcium scores increase with statin treatment, showing a directionally opposite response to clinical benefit [117,118]. Other imaging strategies such as computed tomographic coronary arteriography require the administration of contrast, exposure to radiation, and lack standardized high throughput analyses. While machine learning and artificial intelligence algorithms will doubtless provide an automated and high throughput interpretation of coronary artery computed tomographic angiography, this procedure would still provide a daunting approach to screening for enrollment in a clinical trial.

The recent recognition that clonal hematopoiesis of indeterminate potential (CHIP) increases cardiovascular risk provides a new approach to selecting patients for anti-inflammatory interventions [119]. The common somatic mutations that give rise to CHIP in *DNMT3a* and *TET2* increase components of the inflammasome to IL-β to IL-6 pathway [119,120]. Human genetic studies have shown that individuals with the IL-6 receptor variant associated with reduced IL-6 signaling have reduced cardiovascular events if they have *DNMT3a* or *TET2* CHIP versus those without clonal hematopoiesis [83,121]. This observation supports the concept of selecting individuals for inhibition of the inflammasome–IL-1β–IL-6 pathway components based on CHIP status. Indeed, a preliminary analysis of the minority of individuals in CANTOS for whom genetic consent and DNA were available showed a trend towards greater efficacy of IL-β inhibition in those with *DNMT3a* or *TET2* CHIP [122]. Similarly, CHIP due to *Jak^V617F^* mutations experimentally show activation of the AIM2 inflammasome pathway and reduced thrombotic risk when treated with the Jak1/2 inhibitor ruxolitinib and improvement in indices of plaque stability with IL-1 neutralization [35,123].

In the field of oncology, genotyping for guiding therapy has transformed clinical practice. The targeting of patients with *BCR*-*ABL* translocation chronic myelogenous leukemia with imatinib was approved in 2002. Yet, in cardiovascular medicine we have virtually no interventions that are guided by genotype, save for certain transthyretin mutations. Thus, a way forward for solving the conundrum of clinical trials in cardiovascular medicine in the anti-inflammatory arena could involve targeting anti-inflammatory therapies based on CHIP genotype.

In addition to anti-cytokine therapies and immune therapies described above, the recognition of lipid mediators of resolution of inflammation have provided a possible solution to dissociating alleviating inflammation without impairment of host defenses [124,125,126]. This promising field is poised for clinical testing. Some confusion persists regarding anti-inflammatory versus immunosuppressive therapy. I view agents such as the calcineurin inhibitors or antimetabolites used to mitigate rejection of transplanted organs as immunosuppressive therapies, distinct from anti-inflammatory interventions [127]. They properly target T cell effector mechanisms responsible for organ rejection and carry with them a considerable liability for impaired host defenses. Thus, while fully justified in maintaining solid organ allografts in patients who have advanced disease, the employment of immunosuppressive therapies in chronic atherosclerosis has little appeal given the considerable risk of infection and impaired tumor surveillance. Specialized pro-resolving mediators, small lipid molecules such as the resolvins, maresins, and protectins isolated and characterized by Serhan’s group, promise to inflammation without interfering with host defense mechanisms [124,125,126,127,128].

## 17. Conclusions

The concept of inflammation as a driving force in atherosclerosis encountered considerable resistance in the past. The skepticism likely arose because some initially viewed inflammation as a competing mechanism for atherogenesis over well-established traditional risk factors such as dyslipidemia and hypertension. Yet, the inflammatory hypothesis of atherosclerosis in no way conflicted with traditional risk factors. Rather, inflammation provides a set of pathways by which traditional risk factors may exert their pathogenic action at the level of cells in the arterial wall. We have called this confusion a “false dichotomy” [129]. The success of recent clinical trials targeting IL-1β firmly established anti-cytokine therapy as a new pathway in atherosclerosis therapeutics [130]. The recent trials using colchicine further support the participation of inflammation in the genesis of atherosclerotic events [41,42]. Thus, inflammation in atherosclerosis has progressed from a theory to an established reality [131]. The challenge remains to fine tune anti-inflammatory interventions in a manner that will produce clinical benefit without undue impairment of host defenses. We must meet the challenge of balancing unwanted actions and benefits and deliver agents in a targeted rather than a one-size-fits-all approach to realize the promise of anti-inflammatory therapy for atherosclerosis in the future. A number of the strategies discussed here show the way towards achieving this goal.

## Data Availability

Not Applicable.
